# Supplementary material for: Numerical study on the stress and deformation behavior of buried PE pipeline exposed to stacking load
Source: PLoS One. 2025 Sep 26;20(9):e0329551. doi: 10.1371/journal.pone.0329551 (PMC12469261; doi:10.1371/journal.pone.0329551)
Supplement: Attachment — Submitted filename: Response to Reviewers.docx [file pone.0329551.s002.docx]

**Response to the comments**

**Title:** Numerical study on the stress and deformation behavior of buried PE pipeline exposed to stacking load

**Journal:** PLOS ONE

*We much appreciate the constructive and insightful suggestions made by the editor and reviewers. We have addressed all of the comments, and list below each comment and our response (in italics).*

*Pengcheng Li, Dong Lin, Ming Wen, Fei Wang, Chang Liu, Mingnan Sun, Jianguo Lu**

***According to the comments by Reviewer #1, we have made the following revisions:***

This study presents a numerical investigation into the stress and deformation behavior of buried polyethylene (PE) pipelines subjected to stacking loads. The study develops a coupled soil–pipeline interaction model, validated by laboratory-scale experiments, and explores the effects of various pipeline diameters, burial depths, and loading conditions on mechanical responses. The findings contribute valuable insights into the structural safety and design optimization of buried pipeline systems under surface loading. Overall, the manuscript is logically structured, the results are systematically analyzed. However, certain issues remain, including unclear justification for parameter selection, insufficient methodological detail in the experimental setup. These aspects require revision and clarification to further improve the scientific rigor and practical relevance of the paper.

(1) Redundant phrasing in the Abstract: "buried PE pipeline exposed to stacking load", This phrase is repeated multiple times in the abstract, which affects readability. Rephrase to avoid redundancy.

***Response:*** *Thanks for the good suggestion.* *We have conducted a revision of the abstract to enhance its readability, seen in Lines 20-43.*

***The revised contents:*** *Oil and gas pipelines serve as critical infrastructure for ensuring energy resource supply and security. Stacking load, as one of the most wide-spread loads, frequently acts on the soil surface overlying buried polyeth-ylene (PE) pipelines, influencing stress-strain dynamics and potential fail-ure mechanisms. In this study, a numerical simulation model of pipe-line-soil interaction for buried PE pipelines exposed to stacking load was established and verified. The variations in deformation and stress behavior were systematically analyzed. The results revealed that the maximum de-formation of buried PE pipelines under stacking load occurred at the pipe-line’s center, with upper and lower deformations, as well as diameter vari-ations, diminishing progressively as the distance from the center increases. Furthermore, the disparity between upper and lower deformations was reduced as burial depth was increased. Notably, maximum deformation exhibited a direct proportionality with the stacking load, while both the deformation and its rate of reduction decreased with buried depth in-creased. Additionally, the peak stress within the buried PE pipeline emerged at the center of the load’s footprint, escalating in tandem with the increase in stacking load. Intriguingly, the stress response diverged be-tween pipelines subjected to smaller and larger stacking loads, and the peak stress gradually attenuated as the pipeline’s diameter expands. These findings highlight the importance of considering both stacking loads and buried depths in the design and installation for PE pipelines to safeguard structural integrity and extend operational lifespan.*

(2) In the introduction, it provides a broad review of existing work but does not explicitly clarify the innovation or research gap addressed by this study. Please Add a paragraph at the end of the Introduction stating the gap in previous literature and how your study contributes (e.g., "Few studies have systematically investigated the stress-deformation coupling in buried PE pipelines under varying stacking loads and burial depths. This paper develops...").

***Response:*** *Thanks for the good suggestion. The additional content has been incorporated as per the experts' suggestion. Seen in Lines 121-127.*

***The revised contents:*** *Currently, there is limited research on the stress-deformation coupling mechanism of buried PE pipelines under varying surcharge loads and burial depth conditions. This study establishes a pipe-soil coupling model and derives empirical formulas for pipeline stress in relation to burial depth, as well as pipeline displacement in relation to surcharge load. The findings provide an important theoretical basis and practical guidance for the safe construction and operation of pipelines.*

(3) In Section 2.2, the model validation section does not mention the number of repetitions, sensor resolution, or error margins. Please add the details such as the number of repeated tests, precision of the displacement sensor (e.g., ±0.1 mm), and discuss experimental variability to enhance credibility.

***Response:*** *Thanks for the good suggestion.* *The additional content has been incorporated as per the experts' suggestions. Seen in Lines 211-217.*

***The revised contents:*** *The test was repeated three times to ensure data reproducibility. A LVDT-type displacement transducer with a measurement accuracy of ±0.1 mm was employed as the deformation sensor. The results showed a maximum deviation of 2.3% among the three trials, primarily attributed to slight variations in soil compaction. Nevertheless, the load-displacement curves exhibited highly consistent trends, confirming the reliability of the model.*

(4) The terms “pipe” and “pipeline” are used interchangeably throughout the manuscript. It is recommended to standardize the terminology for clarity and consistency.

***Response:*** *Thanks for the good suggestion. A systematic review of the entire document has been conducted, and the term "**pipeline" has been standardized as the official terminology throughout the text.*

(5) In Equation (3), several symbols such as “τ” are not clearly defined in the text. Please ensure that all variables and parameters (e.g., τ, R, v) are explicitly introduced and described when each equation is presented, to improve clarity and mathematical rigor. In Equation (11), the variable “ri” should be formatted in italic with the “i” as a subscript. Please review and correct the formatting of all equations to ensure consistency with standard mathematical typesetting.

***Response:*** *Thanks for the good suggestion. The format of Equation (3) and Equation (11) has been modified as required. We have reviewed and corrected the formatting of all equations to ensure consistency with standard mathematical typesetting.*

(6) In Section 3, although the content is rich and well-analyzed, the transitions between subsections are not sufficiently smooth. It is recommended to include brief concluding or transitional sentences at the end of each subsection to improve coherence and guide the reader more effectively through the progression of the analysis.

***Response:*** *Thanks for the good suggestion. We have added transitional sentences at the end of Section 3.1 and the beginning of Section 3.2 to enhance the logical flow and better guide readers through the analytical progression.*

***The revised contents:*** *The above results indicate that the displacement of the PE pipeline is positively correlated with the magnitude of the surcharge load, which lays a foundation for further research on its stress variation trends.*

(7) In Table 3, the unit notation for stacking load appears to be incorrect or inconsistent. Please review and correct the unit if necessary. Additionally, it is recommended to check the entire manuscript to ensure that all physical units are accurately and consistently presented.

***Response:*** *Thanks for the good suggestion. We have systematically revised the unit specification for "stacking load" in Table 3, updating it from "N" to "kN/m²". Furthermore, a comprehensive verification and standardization of all physical units throughout the manuscript have been conducted.*

***According to the comments by Reviewer #2, we have made the following revisions:***

In this paper, the stress and deformation behavior of buried PE pipeline exposed to stacking load were studied. The research has some novelty. But there are still parts that need to be corrected before acceptance.

(1) The title should use "deformation" instead of the misspelled "defo-mation".

***Response:*** *Thanks for the good suggestion.* *We have corrected the spelling error in the paper title by revising "defo-mation" to "deformation". Additionally, we conducted a comprehensive review of the entire manuscript to identify and resolve any grammatical inconsistencies.*

(2) The variables in the formulas should be explained and defined upon their first occurrence, with consistent formatting throughout.

***Response:*** *Thanks for the good suggestion.* *We have clearly defined all variables in the equations at their first occurrence and maintained consistent formatting throughout the text.*

(3) The terms "pipe" and "pipeline" in the text should be revised and standardized to one consistent term.

***Response:*** *Thanks for the good suggestion. A systematic review of the entire document has been conducted, and the term "pipeline" has been standardized as the official terminology throughout the text.*

(4) Lines 189-190: The model test box dimensions are stated as height 1.4 m and width 1.5 m, but Figure 2 shows height 1.5 m and width 1.4 m.

***Response:*** *Thanks for the good suggestion. We have corrected the description of the model test tank dimensions and Figure 2 in the text, seen in Lines 196-197.*

***The revised contents:*** *The experimental design and sensor layout was shown in Figure 2, the model test apparatus with a size of 6.0 m×1.4 m×1.5 m (length × width × height) was made by steel plate.*

(5) The coordinate format in Figure 5 needs to be unified, and the units in Figure 8 require superscript formatting. A full review of the manuscript for consistency is recommended.

***Response:*** *Thanks for the good suggestion. We have comprehensively reviewed and revised the coordinate formats of Figure 5 and the units of Figure 8, and conducted a thorough inspection of the figure and table formats throughout the text to ensure compliance with formatting standards.*

***The revised contents:***

*(a) Buried depth of 0.6 m	(b) Buried depth of 0.8 m*

*(c) Buried depth of 1.0 m	(d) Buried depth of 1.5 m*

*(e) Buried depth of 2.0 m*

*Fig. 5 Maximum deformation variation of buried DN50 PE pipeline with different stacking loads*

*(a) 1500 KN/m^2^	(b) 2500 KN/m^2^*

*(c) 5000 KN/m^2^	(d) 7000 KN/m^2^*

*Fig. 8 Stress of DN32 PE pipeline buried at the depth of 0.8 m with different stacking loads*

(6) Line 214: The reference to "Fig. 2b" is invalid as no such figure exists in the manuscript. Please confirm whether this subfigure is missing or requires renumbering.

***Response:*** *Thanks for the good suggestion. The original citation of* "*Fig. 2b* " *on line 214 contained an error that led to misinterpretations during the review process. We have now corrected this reference to* "*Fig. 3b* ". *Seen in Lines 224-227.*

***The revised contents:*** *This congruence underscores the accuracy and reliability of the computational models employed in predicting the deformation behavior of buried PE pipelines under different conditions (Fig. 3b), suggesting that the established buried PE pipeline-soil interaction model can well reflect the variation deformation of buried PE pipeline exposed to stacking load.*

(7) Lines 316-317: The conclusion about correlation should be explicitly supported by data analysis. Please clarify how the correlation was derived.

***Response:*** *Thanks for the good suggestion.* *The derivation of the correlation has been explained as required, with detailed justification provided for the mathematical relationships and assumptions employed in the analysis*. *Seen in Lines 313-320.*

***The revised contents:*** *Through fitting the correlation using different mathematical formulations reveals that a power function aptly captures the relationship between the deformation and the depth of burial, offering a quantitative understanding of this phenomenon,*

$y=c\cdot d_{p}^{d}$ (10)

*where c and d are the fitted parameters, d_p_ is the buried depth acted in the surface of filling soils.*

(8) The 7000 Pa curve in Figure 9 is unclear and requires modification for better visibility.

***Response:*** *Thanks for the good suggestion. We have adjusted the curve line width in Figure 9 from 2 pt to 3 pt to improve visual clarity. Seen in Lines 427-435.*

***The revised contents:***

*(a) DN32	(b) DN40*

*(c) DN50	(d) DN63*

*(e) DN75	(f) DN110*

*(g) DN160*

*Fig. 9 Relationship between the stress and buried depth for the PE pipeline*

(9) The Conclusions section must be revised to align with the updated manuscript. Focus on highlighting the core contributions of the study and ensure the description is concise and focused.

***Response:*** *Thanks for the good suggestion.* *We have revised the conclusion section to ensure alignment with the revisions made throughout the manuscript. Seen in Lines 437-463.*

***The revised contents:***

*This study investigated the variations of deformation and stress of buried PE pipelines via numerical simulations, and some conclusions are drawn,*

*(1) The maximum deformation of buried PE pipeline under stacking loads occurred at the central position. Besides, the upper and lower de-formations, and diameter change decreased with the increase of distance from the pipeline center, particularly for the upper and lower deformations. Additionally, the disparity between upper and lower deformations progressively diminished with greater burial depth.*

*(2) A linear correlation exists between applied stacking load and maximum pipeline deformation. The deformation of buried PE pipeline decreased with the increase of buried depth, as well the rate of deformation reduction. Besides, the deformation-depth relationship could be used to describe by a power function.*

*(3) Peak stress concentrations in buried PE pipelines were localized at the load center. The stress distribution profile transitioned from peaked to flattened with increasing stacking loads. Besides, the stress of pipeline increased with the stacking load raised.*

*(4) For the buried PE pipeline exposed to small stacking loads, the upper stress exceeded the lower stress within the horizontal range of 2.0~4.0 m, while there were little gaps between the upper and lower stresses in the horizon position ranges of 0~2.0 and 4.0~6.0 m. Under heavy stacking loads, the upper stress was higher than the lower stress in the horizon position range of 2.5~3.5m, while reversed results occurred in the horizon position ranges of 0~2.5m and 3.5~6.0m. Furthermore, the peak stress of buried PE pipelines decreased progressively with increasing pipe diameter.*
